# Supplementary material for: Persistent directional growth capability in Arabidopsis thaliana pollen tubes after nuclear elimination from the apex
Source: Nat Commun. 2021 Apr 22;12:2331. doi: 10.1038/s41467-021-22661-8 (PMC8062503; doi:10.1038/s41467-021-22661-8)
Supplement: Supplementary file 1 — Supplementary Information [file 41467_2021_22661_MOESM1_ESM.pdf]

Supplementary Information for

Persistent directional growth capability in *Arabidopsis thaliana* pollen tubes after nuclear elimination from the apex

Kazuki Motomura, Hidenori Takeuchi, Michitaka Notaguchi, Haruna Tsuchi, Atsushi Takeda, Tetsu Kinoshita, Tetsuya Higashiyama, Daisuke Maruyama.

Correspondence to: dmaru@yokohama-cu.ac.jp

This PDF file includes:

Supplementary Figures 1–6
Supplementary Table 1

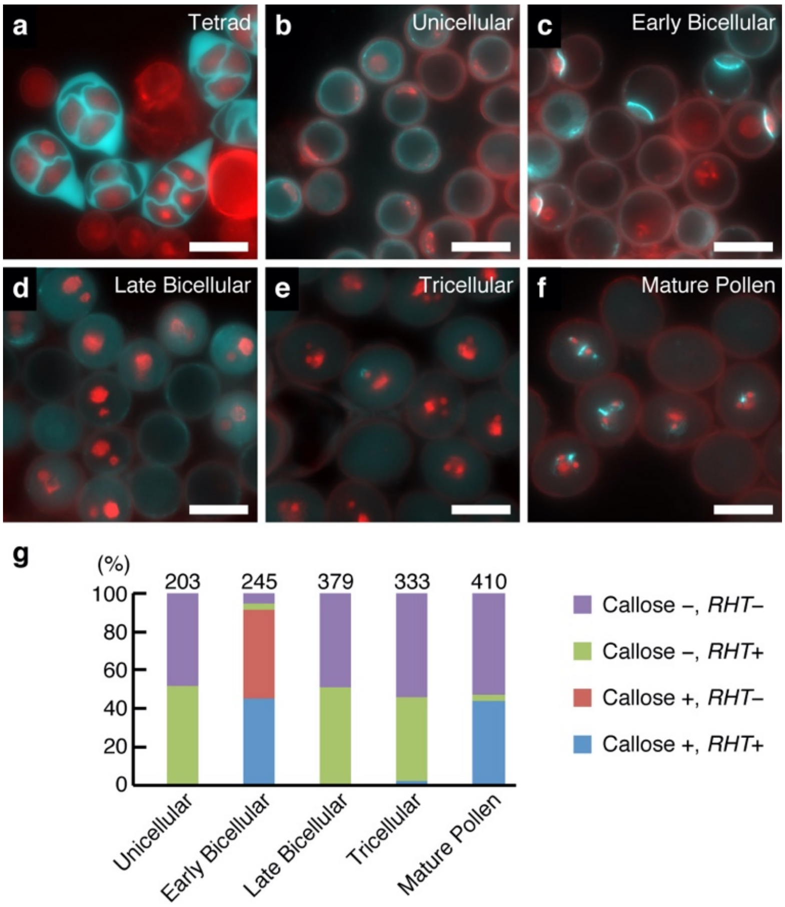

Supplementary Figure 1. Callose deposition during male gametogenesis in a transgenic line harboring *pHTR10:cals3m* and *pRPS5A:H2B-tdTomato*.
a–**f**, Callose deposition (cyan) and patterns of tdTomato-labeled nuclei (red) in various developmental stages of male gametophytes mounted in aniline blue solution. Tetrad stage (**a**), unicellular stage (**b**), early bicellular stage (**c**), late bicellular stage (**d**), tricellular stage (**e**), and mature pollen stage (**f**). Sperm cell-specific callose deposition became obvious after the mature stage. Images (**a) to** (**f)** are representative obtained from two independent observations with similar patterns. **g**, Transition of callose deposition (Callose) during male gametogenesis was analyzed in tdTomato-negative cells (*RHT*−) or tdTomato-positive cells (*RHT*+). The number of microspores observed in each stage is shown above the bar. Scale bars, 20 μm.

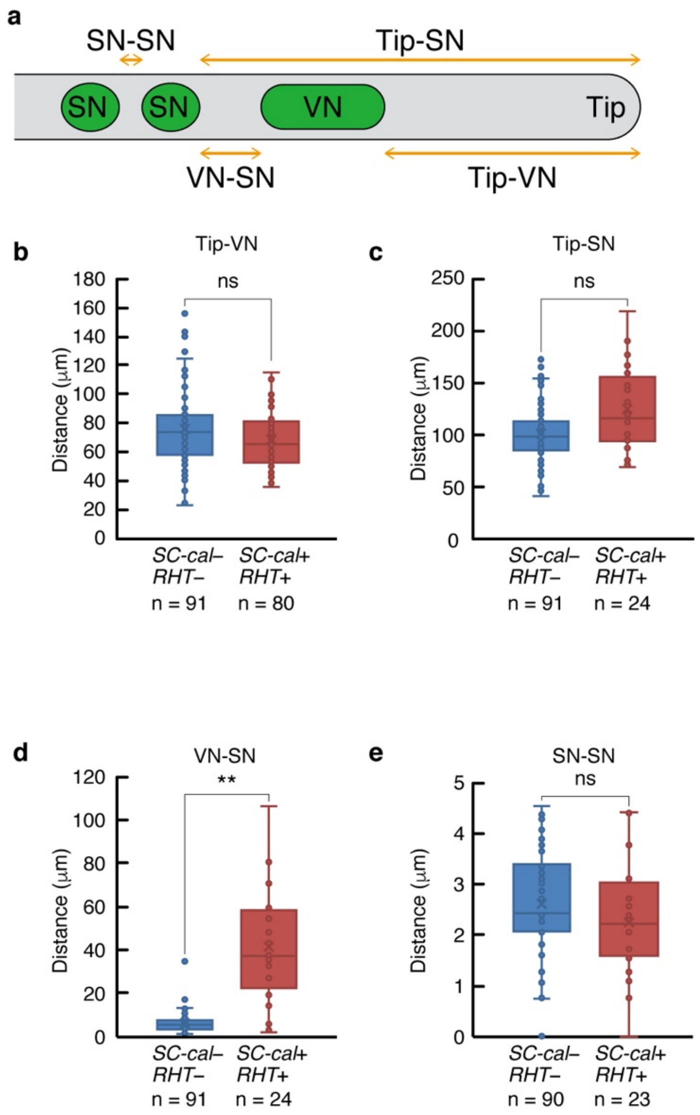

Supplementary Figure 2. Semi-*in vitro* analysis of nuclear positions in pollen tubes from a transgenic line harboring *pHTR10:cals3m* and *pRPS5A:H2B-tdTomato*.
a, Diagram of the apical region of a pollen tube. VN, vegetative nucleus; SN, sperm nucleus. Double-headed arrows indicate distances measured in tdTomato-negative (*SC-cal*− *RHT*−, n = 94) and tdTomato-positive (*SC-cal*+ *RHT*+, n = 81) pollen tubes germinated by the semi-*in vitro* system using SYBR Green I containing medium (see also Fig. 3**c**–**i**). **b**–**e**, Distances from pollen-tube tip to the VN (Tip-VN, **b**), distances from pollen tube tip to the front SN (Tip-SN, **c**), distances from the VN to the front SN (VN-SN, **d**), and distances between two SNs (SN-SN, **e**) in *SC-cal*− *RHT*− and *SC-cal*+ *RHT*+ pollen tubes from the *SC-cal RHT* hemizygous plants. Box-and-whisker plots showing the median (center line), mean (cross mark), upper and lower quartiles (box),

maximum and minimum (whiskers), and points and outlier (circle). P values were calculated by Two-tailed Mann-Whitney U test: $P = 0.03572$ in (**b**), $P = 0.01314$ in (**c**), $P = 1.17 \times 10^{-9}$ in (**d**), and $P = 0.09492$ in (**e**). Asterisks, statistical significance ($P < 0.01$). ns, not significant. The number of pollen tubes measured in each analysis is shown at the bottom. Measured pollen tubes were fewer than 30% in the *SC-cal*+ *RHT*+ in (**c**) to (**e**), since most elongated pollen tubes did not contain sperm cells in the apical region. This characteristic sample population partially explains the lack of significant difference between the *SC-cal*− *RHT*− pollen tubes and *SC-cal*+ *RHT*+ pollen tubes only in the VN-SN (**d**) but not in the Tip-SN (**c**).

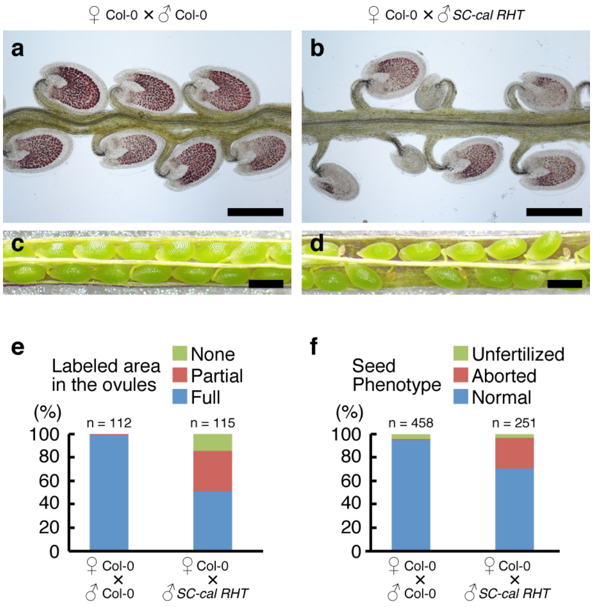

Supplementary Figure 3. Development of seed and seed coats.
a,**b**, Seed coat development of ovules from Col-0 wild-type pistils pollinated by pollen from Col-0 wild-type (**a**) or a transgenic line hemizygous for *pHTR10:cals3m* (*SC-cal*) and *pRPS5A:H2B-tdTomato* (*RHT*) (*SC-cal RHT* hemizygous plants) (**b**). Vanillin staining was performed at 2 days after pollination. Representative images of two pistils (**a**, **b**), nine pistils (**c**), or five pistils (**d**) showing similar patterns. **c**,**d**, Ripening seeds in wild-type pistils pollinated by pollen from wild-type (**c**) or the *SC-cal RHT* transgenic line (**d**). **e**, Percentages of seeds without red staining (None), partially stained seeds (Partial), and fully stained seeds (Full) in **a**,**b**. **f**, Percentages of undeveloped ovules (Unfertilized), small brown seeds (Aborted), and developing green seeds (Normal) in **c**,**d**. This assay used the same *SC-cal RHT* hemizygous plant as that in Figs 2a and 4. Scale bars: 50 μm in **a**,**b**; 0.5 mm in **c**,**d**.

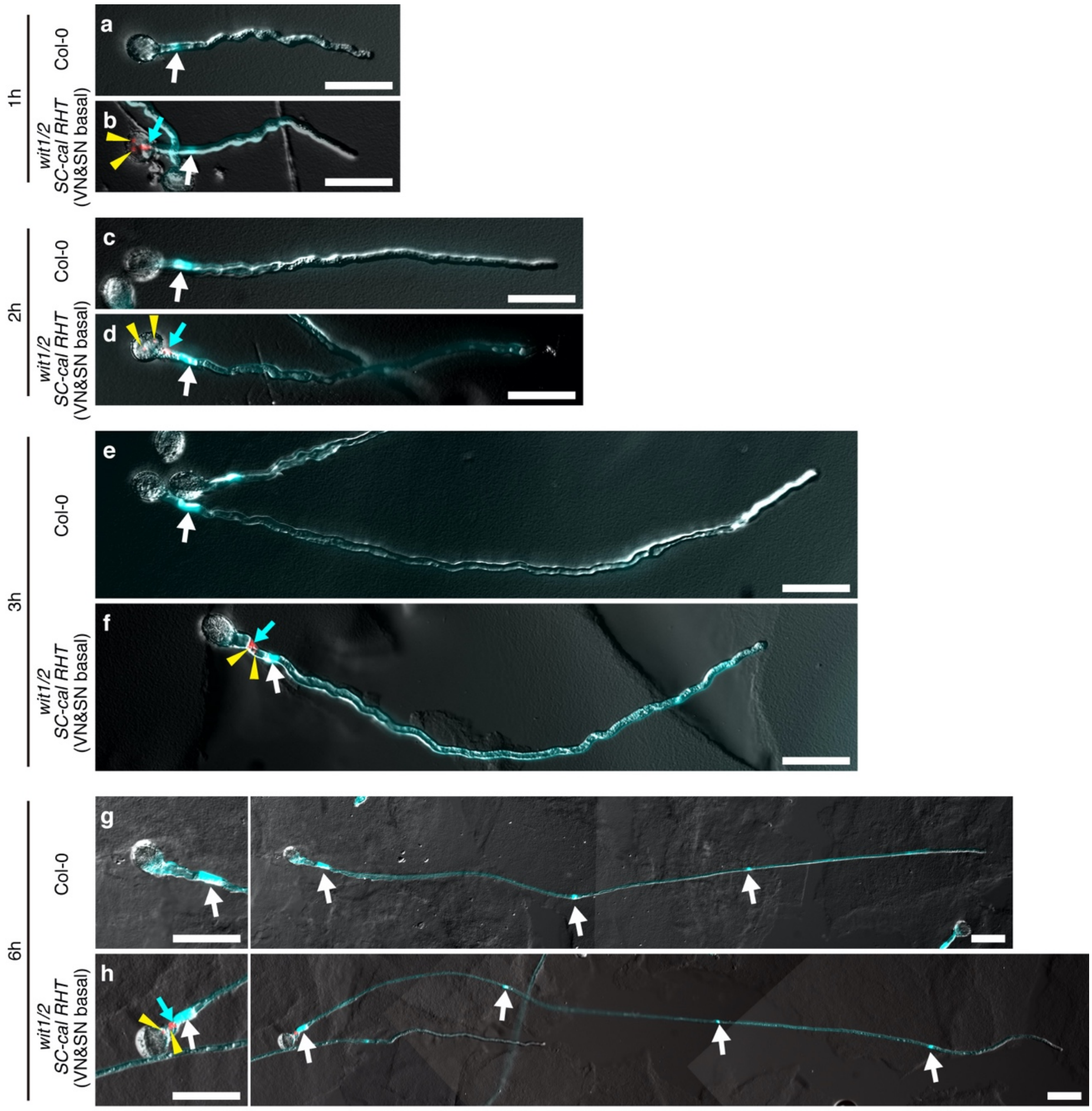

i

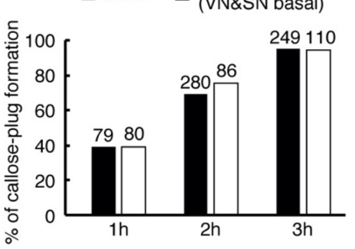

j

Pollen tubes	Callose plugs 1	2	3	4	5	n
Col-0	11.4	26.6	45.5	14.6	1.9	308
wit1/2 SC-cal RHT (VN&SN basal)	12.2	29.9	42.9	12.9	2.0	147

Supplementary Figure 4. Timing and numbers of callose plug formation.
a–**h,** Aniline-blue-labeled pollen tubes from wild-type (Col-0, **a, c, e, g**) or a *wit1 wit2* double mutant hemizygous for *pHTR10:cals3m* (*SC-cal*) genetically-linked with *pRPS5A:H2B-tdTomato* (*wit1/2 SC-cal RHT*, **b, d, f, h**) 1 h (**a,b**), 2 h (**c,d**), 3 h (**e,f**), and 6 h (**g,h**) after incubation on the growth medium. In *wit1/2 SC-cal RHT*, tdTomato-positive pollen tubes that contained two sperm nuclei (yellow arrowheads) and vegetative nucleus (cyan arrows) at the basal region were observed (VN&SN basal). Left panels in (**g**) and (**h**) represent magnifications of the basal part of the pollen tubes shown in each right panel. White arrows: callose plugs. Images in (**a**) to (**h**) are representative of 31 to 237 pollen tubes observed in at least three independent assays. Scale bar: 50 μm. **i,** Percentages of pollen tubes that formed the first callose plug 1 h, 2 h, and 3 h after germination in Col-0 and *wit1/2 SC-cal RHT* (VN&SN basal). Numbers above the bars indicate pollen tubes analyzed. **j,** Percentages of pollen tubes containing one to five callose plugs 6 h after germination in Col-0 and *wit1/2 SC-cal RHT* (VN&SN basal).

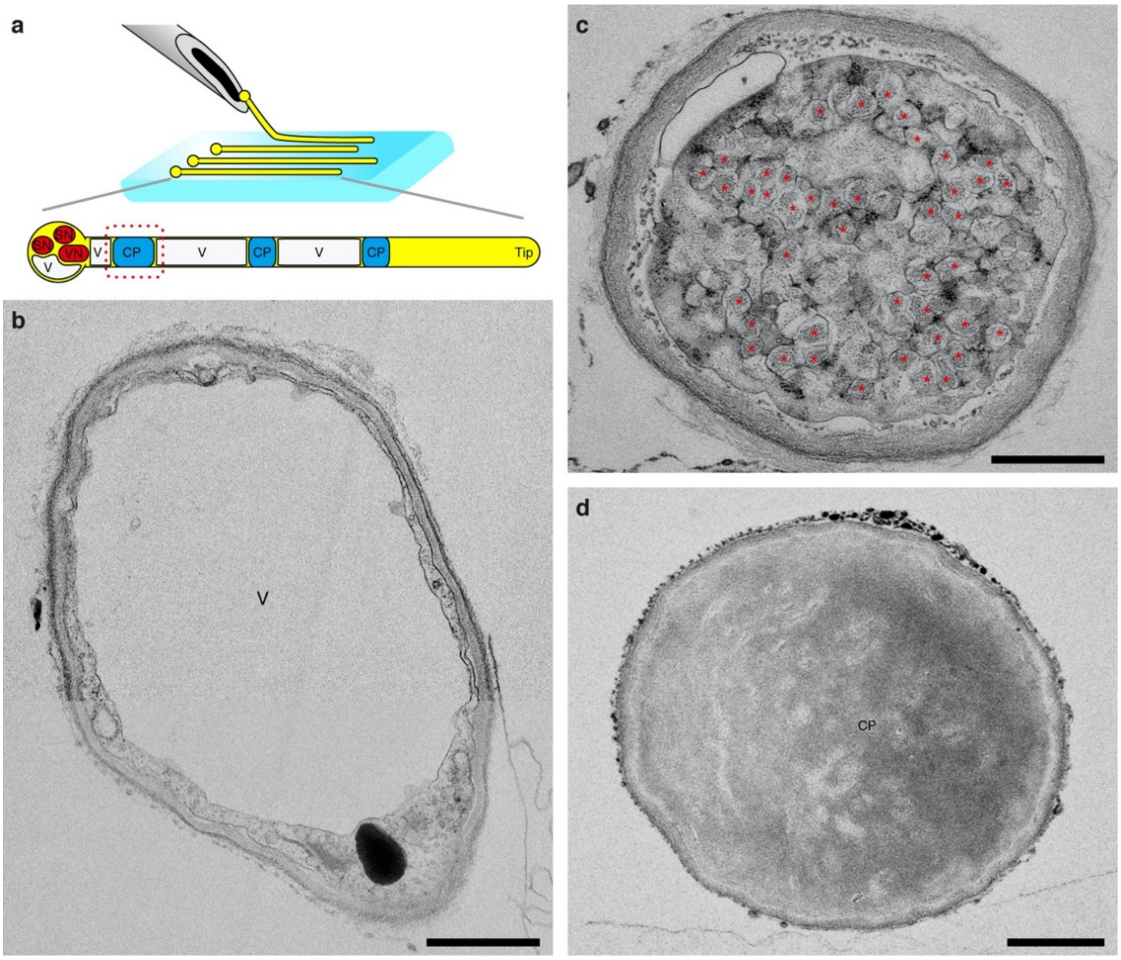

Supplementary Figure 5. Ultrastructural analysis of first callose plug in nuclear-immotile pollen tubes.

a, Diagram of a nuclear-immotile pollen tube and sample preparation for ultrastructural analysis. Pollen from the *wit1 wit2* double mutant hemizygous for *pHTR10:cals3m* and *pRPS5A:H2B-tdTomato* were incubated on pollen tube growth medium for 3 h. Pollen tubes containing immotile sperm cells and vegetative nucleus were dragged out and aligned on fresh medium using a needle under a fluorescence stereomicroscope. Samples were collected from more than three dishes independently prepared, Cross-sections around the first callose plug (red dashed box) were analyzed using transmission electron microscopy. **b,** Area containing a large vacuole. **c,** Callose plug occupied by many callose grains (red asterisks). **d,** Callose plug devoid of vesicular structure. Note that intermediate electron dense material, most likely callose, was fully sealed inside this callose plug. Electron micrographs are representative of more than three sections displaying similar patterns. VN, vegetative nucleus; SN, sperm nucleus; CP, callose plug; V, vacuole. Scale bar: 1 μm.

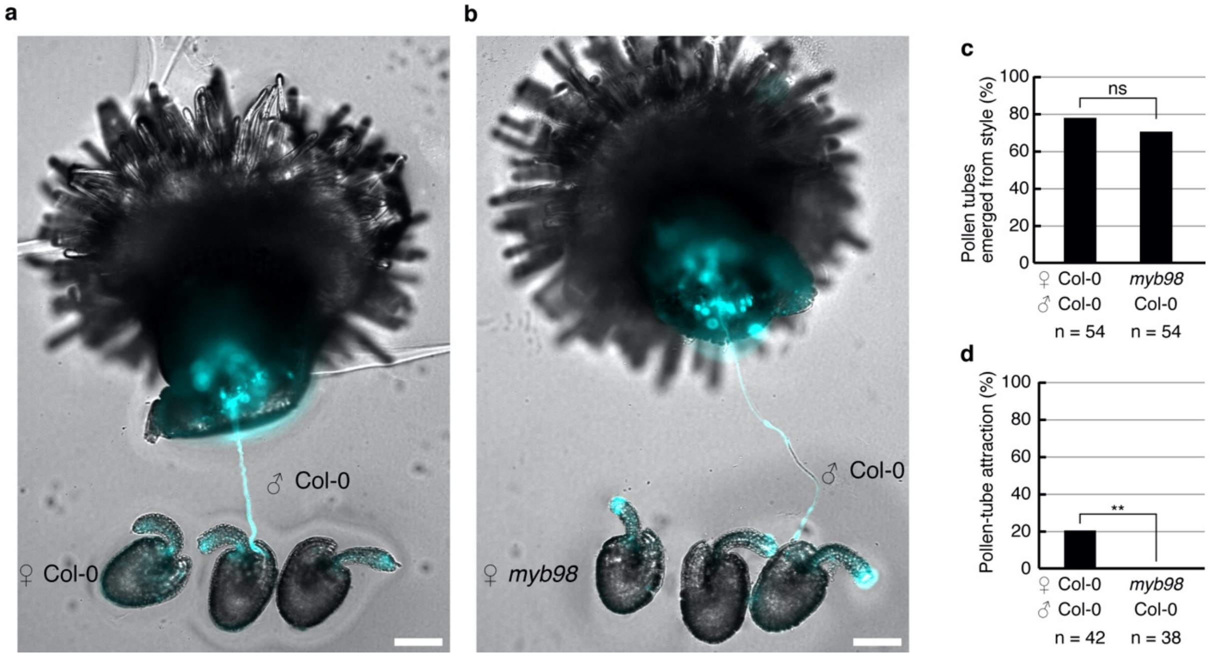

Supplementary Figure 6. Single pollen tube guidance assay in attraction-defective *myb98-1* mutants.

a,b, Representative images of wild-type pollen tubes growing toward Col-0 wild-type ovules (Col-0, **a**) or attraction-defective *myb98-1* mutant ovules (*myb98*, **b**) visualized by aniline blue staining after the single pollen tube guidance assay (see schematics in Figure 8a). Representative images of 11 (**a**) or 38 (**b**) independent assays showing similar patterns. **c**, Percentages of pollen tubes that emerged from the cut-style (Chi-square test, p = 0.38). **d**, Percentages of pollen tubes that entered the micropyle in the ovules (Chi-square test, P = 0.000682). Scale bar: 100 µm.

Supplementary Table 1 Primer list used in this study.

Primer name	Sequence (5`-3`)
pHTR10HindIII_F	GCGAAGCTTACTTCTCCGACCAAAAACTTTCAAAATTTC
pHTR10HindIII_R	GCGAAGCTTCTTCGAGAGAACGATGATGATGATGATAAC
pLAT52HindIII_F	GCGAAGCTTATACTCGACTCAGAAGGTA
pLAT52HindIII_R	GCGAAGCTTAAATTGGAATTTTTTTTTTTGGTGTGT
pACA3HindIII_F	CGGCCAGTGCCAAGCTTTAGGTTTCCTTAATT
pACA3HindIII_R	TGCAGGCATGCAAGCTTCTTTTGTTTTTTCTT
FP_SpeI_F	TGAACTAGTAAGGGTGGGCGCGC
FP_EcoRI_R	CATGAATTCGCTGCCGCCGCTG
SYP132_EcoRI_F	AGCGAATTCATGGACGATCTTCTGAAGGG
SYP132_SpeI_R	CTTACTAGTTCAAGCACTCTTGTTTTTC
Lyn24-mNG_F	ATGGGATGTATCAAATCTAAACGAAAGGATAACCTCAATGATGACGAAGTA
	GATTCAAAAACTCAACCGGTTATGGTGAGCAAGGGCGAG
FP_R	TTACTTGTACAGCTCGTCCATGCC
pENTR_CFP_F	CACCATGGTGAGCAAGGGCGAGGAGC
RANGAP1pro_F	GTCGACTCTAGAGGATCCTTCTCCAACGAATCTGCAATG
RANGAP1_R	GGACAATGGTACCACATCTTCCTCCCCTTGCTTGATTTTC
Linker1_FP_F	GATGTGGTACCATTGTCCATGGTGAGCAAGGGCGAGGAA
mNG_R	GGCCGCAGCTCCGGACTTGTACAGCTCGTC
