# Supplementary material for: Persistent directional growth capability in Arabidopsis thaliana pollen tubes after nuclear elimination from the apex
Source: Nat Commun. 2021 Apr 22;12:2331. doi: 10.1038/s41467-021-22661-8 (PMC8062503; doi:10.1038/s41467-021-22661-8)
Supplement: Supplementary file 3 — Descriptions of Additional Supplementary Files [file 41467_2021_22661_MOESM3_ESM.pdf]

Descriptions of Additional Supplementary Files

Supplementary Movie 1
Description: Nuclear dynamics of pollen tubes with or without callose accumulation in sperm cells. Time-lapse imaging of semi-in vitro-germinated pollen tubes from a double transgenic plant carrying pHTR10:cals3m (SC-cal) genetically-linked with pRPS5A:H2B-tdTomato (RHT) (SC-cal RHT hemizygous plants). Pollen tubes were grown in medium supplemented with SYBR Green I. In the SC-cal-absent wild-type pollen tube, the nuclear triplet did not have tdTomato signals (RHT−, green) and exhibited normal apical transport. However, in pollen tubes carrying the SC-cal, tdTomato-labeled vegetative nucleus and sperm nuclei (RHT+, magenta, or white) appeared to show independent movements.

Supplementary Movie 2
Description: Analysis of the connection between the vegetative nucleus and sperm cells in the pollen tubes. Time-lapse imaging of semi-in vitro-germinated pollen tubes from a transgenic plant hemizygous for pHTR10:cals3m (SC-cal) genetically-linked with pRPS5A:H2B-tdTomato (RHT) and homozygous for the pRANGAP1:RANGAP1-mNeonGreen nuclear envelope marker and pHTR10:mRUBY2-SYP132 sperm cell-specific plasma membrane marker. A connection between the vegetative nucleus and sperm cells was visible in the tdTomato-negative SC-cal-absent pollen tube (RHT−), while the tdTomato-positive SC-cal-present pollen tube (RHT+) had no such connection.

Supplementary Movie 3
Description: Nuclear dynamics of mutant pollen grains before and after germination. Time-lapse imaging of in vitro-germinated pollen from a wit1 wit2 double mutant carrying pHTR10:cals3m (SC-cal) genetically-linked with pRPS5A:H2B-tdTomato (RHT). Confocal Z series images were captured every 2 min in pollen grains containing tdTomato-labeled sperm nuclei (SN) and vegetative nucleus (VN). In the upper movie, the VN entered the pollen tube, but the SNs stayed in the pollen grain (VN apical, SN basal). However, all three nuclei remained in the lower movie (VN&SN basal). Onset of pollen-tube germination shown at 0 min. The numbers in the movie indicate cases of each pollen.

Supplementary Movie 4
Description: Time-lapse movies of photobleached pollen tubes. Photobleaching of blue fluorescence at the apical half of in vitro-germinated pollen tubes from a wit1 wit2 double mutant hemizygous for pHTR10:cals3m (SC-cal) genetically-linked with pRPS5A:H2B-tdTomato (RHT) and hemizygous for pLAT52:mTurquoise2. The first and second movies indicate pollen tubes before or after callose formation whose sperm cells and vegetative nucleus were isolated in the basal region. Blue fluorescence of mTurquoise2 was stable at the basal region in the pollen tube with a callose plug. Time-lapse images of pre- and post-bleaching were captured at 15 s intervals. Each set of photobleaching took ~10 s.
